# Reduced Biodiversity is Linked to Higher Triatomine Occupancy: Chagas Disease Implications

**DOI:** 10.1007/s10393-025-01729-7

**Published:** 2025-06-27

**Authors:** Maya Rocha-Ortega, Alex Córdoba-Aguilar

**Affiliations:** Instituto de Ecología, Universidad Nacional Autónoma de México, Apdo. Postal 70-275, Circuito Exterior, Ciudad Universitaria, 04510 Coyoacán, Ciudad de Mexico Mexico

**Keywords:** Dilution effect, Kissing bugs, NDVI, Occurrence

## Abstract

The hypothetical link between a reduction in biodiversity and increased zoonotic diseases remains debatable. We tested this by utilising remote vegetation density and protected areas (PAs) as proxies for biodiversity and examined their relationship with triatomine richness and occupancy. Triatomine bugs serve as the primary vector for *Trypanosoma cruzi*, the causative agent of Chagas disease. Although no link was found between triatomine species richness and vegetation, triatomine occupancy was lower within PAs than outside. Consequently, our findings suggest that reduced biodiversity may lead to a greater occupancy of triatomines, which could increase the risk of Chagas disease.

**Supplementary Information:**

The online version contains supplementary material available at 10.1007/s10393-025-01729-7.

One major challenge to humanity is to identify the origins and contributing factors that underlie zoonotic diseases (Allen et al. 2017). A related idea is that deforestation leads to a greater and non-uniform spatial prevalence of vector-borne pathogens (da Xavier et al. 2012; Burkett-Cadena and Vittor 2018). This phenomenon implies that more diverse ecological and stable communities help to mitigate the spread of diseases, a hypothesis known as the dilution effect (Civitello et al. 2015). According to this, zoonotic diseases, for example, will exhibit a higher prevalence in areas with lower biodiversity (Halliday et al. 2020).

Protected areas (PAs) provide a good framework to examine the dilution effect hypothesis. Biodiversity levels of PAs, as opposed to non-PAs, are significantly higher with lower rates of landscape conversion and stable or increasing trends in wildlife populations (Pringle 2017). Moreover, regarding diversity of insect vectors and PAs, the evidence remains inconclusive. The following example illustrates this: While the abundance of tsetse flies increases within PAs (Lord et al. 2018), this is not the case for mosquitoes of medical importance (Davidson et al. 2024).

Chagas disease (CD) is caused by *Trypanosoma cruzi*, an obligate parasite with a complex life cycle: Kissing bugs (Reduviidae: Triatominae) are the primary host while terrestrial mammals are the secondary host (Ibarra-Cerdeña et al. 2017). Kissing bugs exhibit low species-host specificity (Izeta-Alberdi et al. 2016), and their richness and occupancy are more correlated with socioeconomic than environmental factors (Rocha‐Ortega et al. 2024).

Satellite remote sensing is an instrumental tool for capturing variation in vegetation and landscape structure at fine spatial resolutions along large spatial extents (Ribeiro et al. 2019). For instance, the Normalised Difference Vegetation Index (NDVI) measures the density of plant matter—using the near-infrared and visible red wavelengths (Lord et al., 2018). One application of NDVI is to map vector-borne diseases with prominent advances over the past 40 years, mainly to look for vector habitats and potential control of mosquitoes, ticks, blackflies, tsetse flies, and sandflies (Kalluri et al. 2007).

We have investigated the potential relationships among the NDVI, PAs, triatomine species richness, and occupancy. In accordance with the dilution effect hypothesis, we predicted that both triatomine species richness and occupancy would be greater outside PAs and with lower NDVI values. The aforementioned predictions are predicated upon a) an amplified presence of zoonotic reservoirs for triatomine species (Gibb et al. 2020) and b) a heightened abundance of triatomines outside ANPs attributable to an increased number of triatomine shelters (e.g. *Attalea* palms, human residences, etc.) (Ribeiro et al. 2015; Rodriguez and Loaiza 2017) along with a reduced species richness of wildlife mammals (da Xavier et al. 2012).

We compiled species occurrence records for triatomines in the Americas to ascertain these relationships using DataTri information (Ceccarelli et al. 2018) and Villalobos et al. (2019). Next, we removed duplicate records and employed only the records from 1981 to 2016, because this period is the most complete in collections of insects (Rocha-Ortega et al. 2021). We utilised the NDVI values published by Pinzon et al. (2023). Besides, we used polygons of protected areas published by WDPA (UNEP-WCMC 2024). Then, we utilised the Global Terrestrial Ecoregions (https://geospatial.tnc.org/). Therefore, we utilised species richness measured as the sum of species within a unique latitude and longitude coordinate for each year, and assigned an NDVI value to each point corresponding to the year of collection. Next, we calculated the distance from the record point to the closest protected area polygon limit. Likewise, we assigned an ecoregion to each record point. With these data, we applied the Bayesian Generalised Linear Mixed-Effects Model with a Poisson distribution to relate species richness with NDVI values and distance to PAs, using ecoregion as a random variable (see Fig. 1). We used the function *bglmer* from the blme package in R (R Development Core Team 2017). We inspected usual goodness-of-fit measures, using the function *Anova* from the car package, over/underdispersion using the *dispersion_glmer* function from the blmeco package, collinearity using *check_collinearity* from performance package, and plot the residuals. The model showed low dispersion, collinearity, and good fit to the distribution.

**Figure 1:**
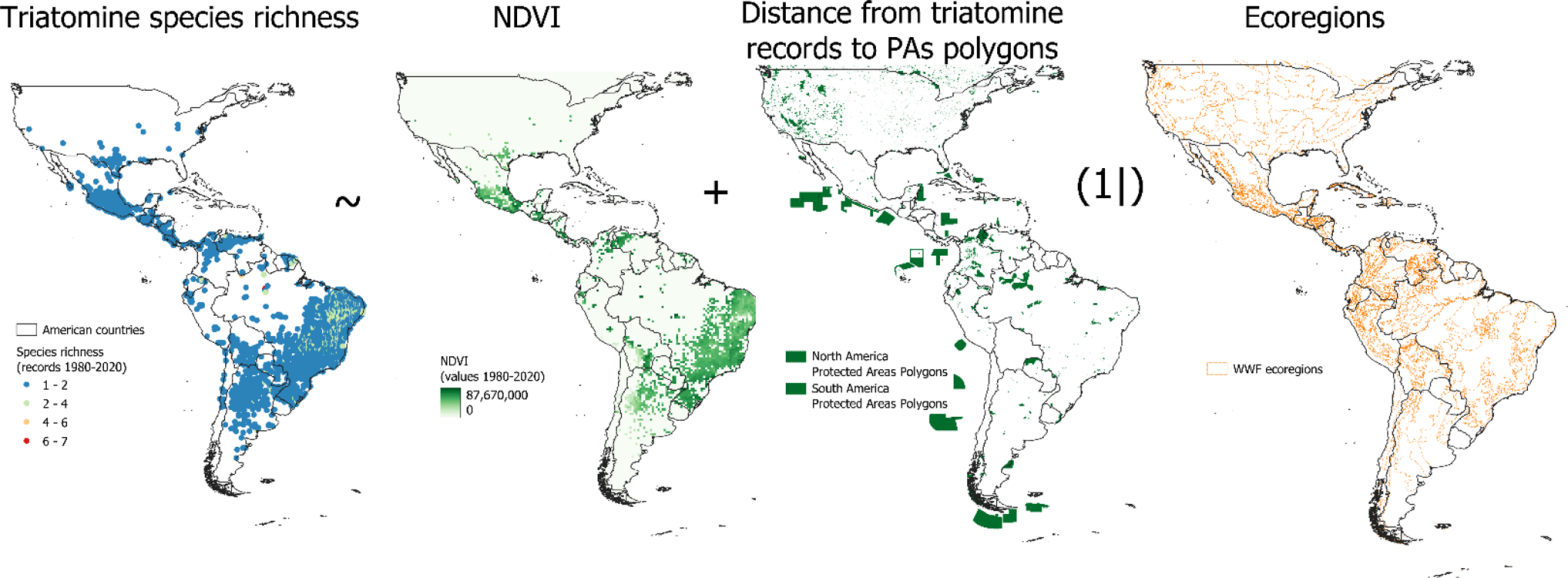
Exemplification of the Bayesian generalised linear mixed-effects model.

Finally, to test the probability of triatomines inside or outside PAs, we created an empty map of 0.5° × 0.5° grids along the triatomine distribution in America. Thereafter, we assigned a value of 1 to grids with triatomine records or PAs and 0 if not. The probability that triatomine occupancy inside or outside the PAs was calculated using a Multivariate Generalised Linear Mixed Model with an ordinal distribution, utilising the ecoregion as a random variable (see Fig. 2). We used the *MCMCglmm* function of the MCMCglmm package, and the analysis was conducted in R (R Development Core Team 2017).

**Figure 2:**
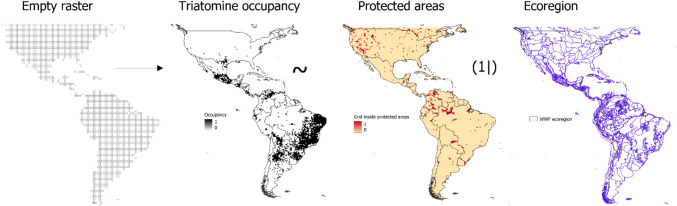
Exemplification of the multivariate generalised linear mixed model.

We found that the relationship between triatomine species richness with NDVI (χ^2^ = 0.006, *p* = 0.62) and distance to a protected area (χ^2^ = 0.008, *p* = 0.66) was not significant. Furthermore, we found that the probability of triatomine presence is lower inside the protected areas than outside (post. mean =  − 0.40, ICL =  − 0.65, ICU =  − 0.19, *p* < 0.001). NDVI was not significant in explaining triatomine species richness. In relation to this, we assumed that all triatomine species would respond similarly to vegetation density. Moreover, Ibarra-Cerdeña et al. (2009) asserted that three taxonomic complexes are associates with open habitats and two with wooded habitats in North America. This suggests that high NDVI values benefit some species but negatively impact others, negating the trend between triatomine species richness and vegetation density. Furthermore, areas with a higher density of plant matter could support more diverse mammalian communities, resulting in intra- and interspecific competition among triatomines (Zacharias et al. 2017), likely constraining their species richness. Actually, our findings suggest that this phenomenon occurs because among 2161 sites, 2132 are species-poor communities, and only one site reported seven species.

Inside PAs, the probability of triatomine occupancy was lower inside than outside PAs. We assumed that triatomine community predominantly exists outside ANPs: Only two of the 98 species examined were collected exclusively within ANPs, 75 species outside, and 21 species in both environments (Table SM1). This pattern is likely due to increased species abundance outside of ANPs, a phenomenon corroborated by various studies (Gottdenker et al. 2011; Rodriguez and Loaiza 2017; Ocaña-Mayorga et al. 2018). As a matter of fact, prevalence and load of *T. cruzi* infection among host species are significantly greater outside protected areas than within (Orozco et al. 2016), which is attributed to a decreased mammalian species richness (da Xavier et al. 2012). Therefore, our study, in conjunction with other research, supports that protection categories influence the transmission cycles of the parasite. Note, however, that the vector transmission variation depends on spatial scale (Lord et al., 2018). The dilution effect hypothesis supposes that at a smaller spatial scale; it is more likely to find a negative relationship between biodiversity and the likelihood of zoonotic diseases (Rohr et al. 2020). We do not discard this for the case of our study, but it would be necessary to identify local key habitats with high mean and variance of infestation to assess this (Cohen et al. 2017).

Therefore, our results are only partially consistent with the dilution effect hypothesis, which suggests that PAs could reduce the probability of Chagas disease occupancy. In any case, health authorities may use our findings to increase vigilance and control programmes in areas with reduced biodiversity.

## Supplementary Information

Below is the link to the electronic supplementary material.

Supplementary file1 (CSV 4 KB)
